# Supplementary material for: Biophysical simulations of fMRI responses using realistic microvascular models: Insights into distinct hemodynamics in humans and mice
Source: Imaging Neurosci (Camb). 2026 Sep 8;4:IMAG.a.1340. doi: 10.1162/IMAG.a.1340 (PMC13556804; doi:10.1162/IMAG.a.1340)
Supplement: Supplementary file 1 [file IMAG.a.1340_supp.pdf]

Biophysical simulations of fMRI responses using realistic microvascular models: insights into distinct hemodynamics in humans and mice

Grant Hartung[1,2], Avery J. L. Berman[1,2,3,4], Sava Sakadžić[1,2], Andreas Linninger[5,6], David A. Boas[7], Jonathan R. Polimeni[1,2,8,†]

[1]Athinoula A. Martinos Center for Biomedical Imaging, Massachusetts General Hospital, Charlestown, MA, USA.
[2]Department of Radiology, Harvard Medical School, Boston, MA, USA.
[3]Department of Physics, Carleton University, Ottawa, ON, Canada
[4]Institute of Mental Health Research, Royal Ottawa Mental Health Centre, Ottawa, ON, Canada
[5]Department of Bioengineering, University of Illinois at Chicago, Chicago, IL, USA.
[6]Department of Neurosurgery, University of Illinois at Chicago, Chicago, IL, USA.
[7]Neurophotonics Center, Department of Biomedical Engineering, Boston University, Boston, MA, USA.
[8]Harvard-MIT Program in Health Sciences and Technology, Massachusetts Institute of Technology, Cambridge, MA, USA.
[†]*Current address:* Richard M. Lucas Center for Imaging, Department of Radiology, Stanford University, Stanford, CA, USA.

Supplementary Notes

1. Validation with awake mouse data

In addition to the dynamic oxygenation validations presented in the main text based on recordings from anesthetized mouse (see **Figure 3** in the main text), we also compared our simulations against newer data recorded in the awake mouse (Şencan et al., 2022). These results are shown below in **Supplementary Figure S1**. While we observed a close agreement between the experimentally measured pO_2 responses with our simulated responses in the veins, we observed a poor agreement in arteries. We do note, however, a close agreement in the simulated pO_2 values using both the previous oxygenation simulation framework from Fang & Boas and our proposed oxygenation solver.

The discrepancy between the measured and simulated oxygen dynamics in arteries may be attributable to the boundary conditions used in our simulations. Due to our fixed (unvarying) inlet concentration of oxygen and fully saturated blood at the inlet (i.e., and SO_2 of approximately 100%), the simulated arterial segments are simply not capable of strong changes in oxygenation. This could potentially be overcome by dynamically adjusting our boundary condition (e.g., by enabling a rise in arterial inlet pressure and oxygenation during activation), however because we do not possess experimental data describing this dynamic change in pressure we did not attempt to extend our model in this way. Moreover, at this time, we do not have a biophysical explanation for the experimental observation of larger change in the arterial oxygen compared to venous oxygen measured during activation (Şencan et al., 2022), therefore more experimental data may be needed to fully explain these measurements.

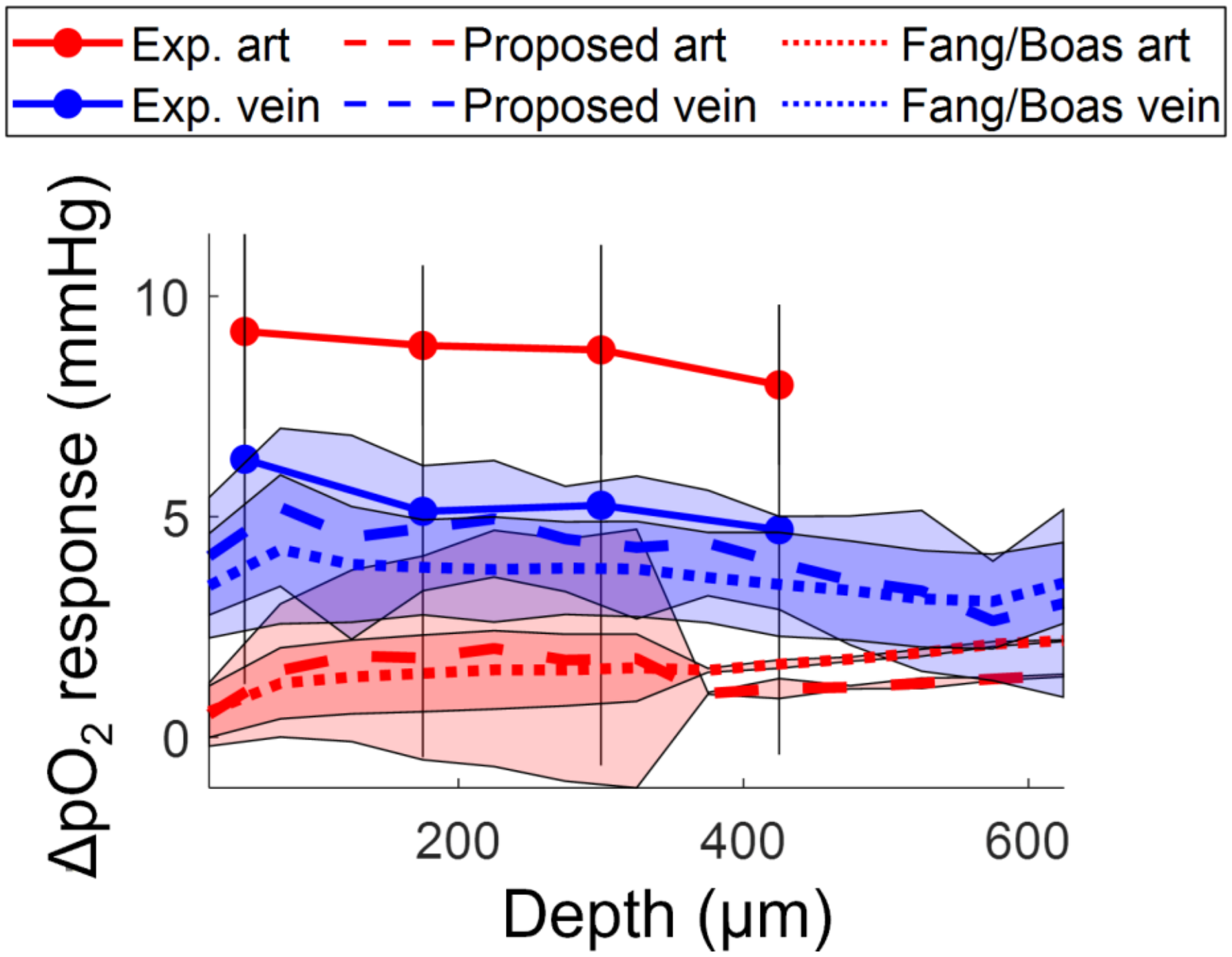

Supplementary Figure S1. Validation of dynamic oxygen simulations against awake mouse imaging. We overlaid the active oxygen tension response in arteries (red) and veins (blue) for three datasets, binned by cortical depth. We show experimentally measured oxygen tension (solid lines), simulated oxygen tension using the previous gold-standard method for similar oxygenation calculations, termed the "Fang/Boas" method (dotted lines) and using our proposed method (dashed lines). Data and simulations from arteries are depicted in red and from veins are depicted in blue.

2. Dynamics of $CMRO_2$ response with a 1st-order rate constant

Previous biophysical modeling approaches simulate $CMRO_2$ by estimating the reaction rate of oxygen with a kinetic equation using a 0th-order reaction rate constant (Fang et al., 2008; Gagnon et al., 2015). This is equivalent to assuming that the rate of oxygen metabolism is independent of the local concentration of oxygen. Our modeling framework instead implements a more realistic 1st-order reaction rate constant, under which the rate of oxygen metabolism is a function of available oxygen, which results in, e.g., oxygen depleted regions undergoing less oxygen consumption. This choice of a 1st-order reaction rate is motivated by the improved mathematical stability it provides (i.e., it helps avoid negative oxygen concentrations in vessel-sparse regions). This model is also biologically inspired in that regions of lower vascular density and thus lower oxygen delivery likely reflect reduced neuronal density (Blinder et al., 2013) in regions with higher density which ostensibly consume less oxygen. This means that, at baseline, oxygen consumption will reflect oxygen availability, and during dynamic responses to neuronal activation and the corresponding functional hyperemia, oxygen consumption will increase with blood flow and oxygen delivery.

For simplicity, our model implements a 1st-order reaction rate by computing the equivalent 0th-order reaction rate constant (R_0) that would yield the identical total oxygen metabolism as a 1st-order model as described by the definition

$$R_0 = \frac{\sum k \, c_{02}}{V} \quad (1)$$

where V is the domain volume and c_{02} is the concentration of oxygen in each tissue element. The product of the reaction rate coefficient k with oxygen concentration in each element c_{02} represents the consumption in each tissue element, and the sum across all elements gives the total metabolism rate of the entire voxel. We then set the value of k to achieve the target OEF as described in the main manuscript. This model has the advantage over a 0th-order model because it does not over-consume in areas depleted of oxygen (an effect that would lead to negative oxygen levels in parts of the tissue). It is also simpler than other models such as the Michaelis-Menten reaction model that was designed for diffusion-limited enzyme-catalyzed reaction systems.

As oxygen delivery increases during neuronal activation (i.e., during functional hyperemia), the 1st-order reaction rate implemented in our model will result in an increased metabolism, corresponding to a higher R_0 value. In other words, the consumption of oxygen is proportional to the delivery of oxygen, which follows the flow and the active arterial dilation responses in the hemodynamic simulation. As an example, the estimated R_0 value for rVAN1 described in the main text is 2.2 μmol/ml/min at baseline and 2.5 μmol/ml/min during activation, an increase of 12.6%. These values are in line with past reports of 0.25–2.96 μmol/ml/min (see **Supplementary Table S2**) and a 10% change in $CMRO_2$ from literature (Buxton et al., 2004). The time-course of the dynamic oxygen consumption, or the $\Delta CMRO_2$ response to neuronal activity, is shown in **Supplementary Figure S2**. The result is a dynamic

oxygen consumption response that closely follows the dynamic blood flow response; classic BOLD fMRI models such as the Balloon Model framework similarly assume that the flow and oxygen consumption dynamics follow similar dynamics (Buxton et al., 2004). In our model, the dynamic oxygen consumption is time-locked to the blood flow does not alter the temporal features of the BOLD response but it does cause a change in the BOLD response amplitude.

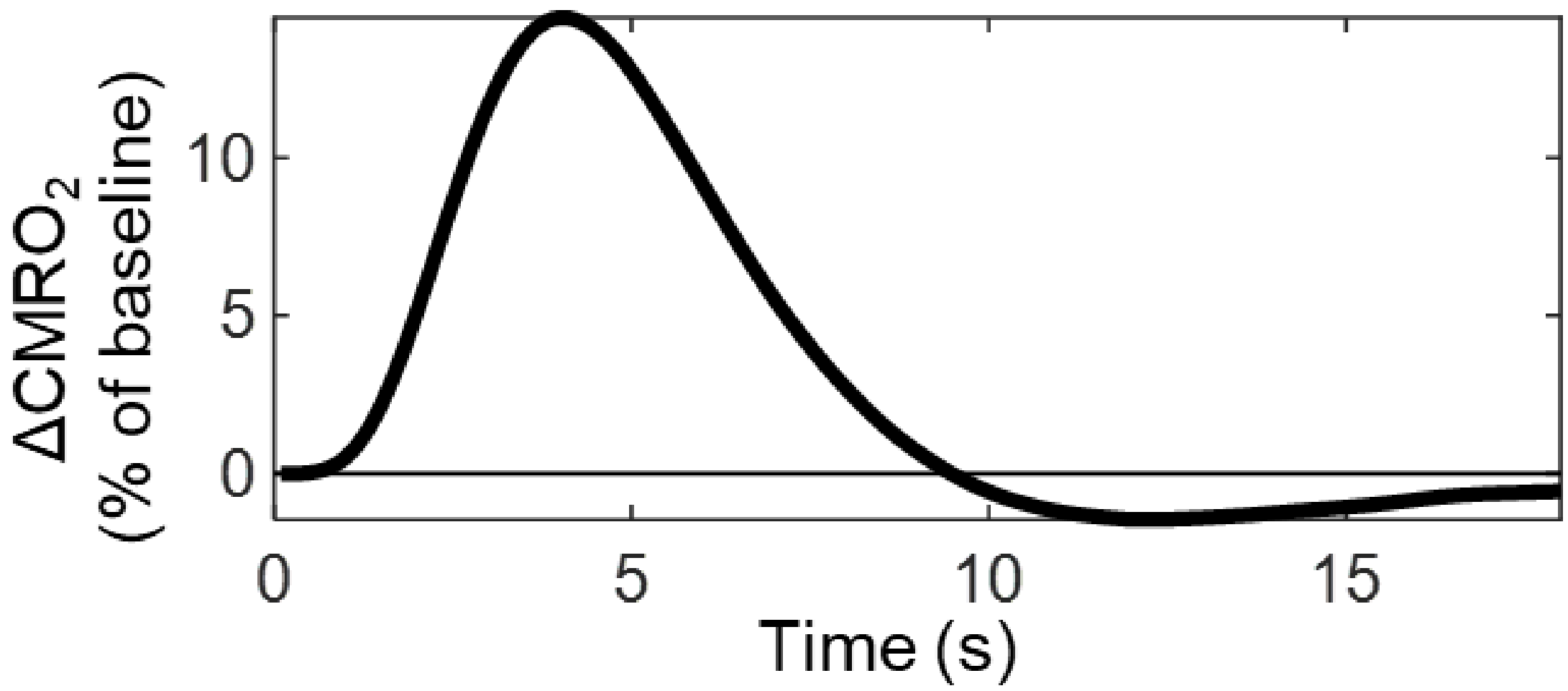

Supplementary Figure S2. Dynamic $CMRO_2$ response to neuronal activity, provided by the 1st-order reaction rate model of oxygen consumption. Here $\Delta CMRO_2$ is plotted for rVAN1 in response to neuronal activity. Under our oxygen consumption model, as blood flow (and thus oxygen delivery) increases or decreases, the oxygen consumption follows, resulting in a time-course similar to that of the hemodynamic response. In this example, $\Delta CMRO_2$ peaks at 14.4% relative to baseline.

3. BOLD initial dip appeared with some VANs but not others

The BOLD simulations based on some of our VAN models generated a noticeable "initial dip" or transient reduction in the BOLD response roughly 1–2 s after stimulus onset followed by the main positive BOLD response (see **Figures 5C**, **6E** & **7** in the main text). Notably, the BOLD responses based on reconstructed mouse VANs exhibit an initial dip whereas the BOLD responses from synthetic mouse VANs did not. Because the initial dip is well known to be 'elusive' and not routinely observed in *in-vivo* fMRI data (Buxton, 2001), we further investigated the cause of the BOLD initial dip in our simulated fMRI data. We reasoned that the presence or absence of a BOLD initial dip may be attributable to the number of terminal arteries and veins supplying and draining the VAN model, and these numbers do vary across our VAN models. We hypothesized that the greater numbers of terminal arteries and veins in rVANs would result in slower baseline blood velocity, leading to the presence of a BOLD initial dip, as explained in the *Discussion* section: during initial dilation, blood outflow of expanding vessels will transiently decrease in order to fill the expanding volume (i.e., the "transverse blood flow" phenomenon, described in the original "Windkessel" BOLD fMRI model (Mandeville et al., 1999)) which leads to longer blood residency time, increased OEF, and a dip in blood oxygenation in downstream vessels.

As a first step in testing this hypothesis, we simulated the BOLD response in a rVAN that exhibits an initial dip and examined the BOLD signal within each individual vascular compartment (i.e., arteries, veins, and capillaries) independently. To perform this, we used the volume and oxygenation time-courses from the dynamic response of the entire VAN model, as described in the *Online Methods* section. To isolate the compartments, we simulated the BOLD response for each compartment individually, while including the baseline values (of volume and oxygenation) for the inactive compartments. We observed that the BOLD initial dip generated using the reconstructed VANs was isolated to the capillary compartment (**Supplementary Figure S3**). Experimental data in mouse somatosensory cortex (Tian et al., 2010) demonstrated that the BOLD initial dip is most prominent at the pial surface and is unnoticeable in the capillaries, suggesting an apparent disagreement between our simulations and the empirical data.

We hypothesized that this BOLD initial dip is generated in certain VANs by slow velocity in the affected vessels, which appear to be the capillaries. This may be caused by specific structural features in those VAN models combined with the slow capillary blood velocity—i.e., rVANs with more arterial inlets and venous outlets will exhibit slower arterial and capillary baseline velocity. To test this hypothesis, we manipulated a synthetic VAN model that originally did not produce a BOLD initial dip to reduce the baseline velocity in the capillaries. Specifically, we increased the baseline vessel diameter of the capillary segments of sVAN1 while maintaining the same volumetric flow rate (same overall perfusion). We observed that by expanding the capillary diameter at baseline by 2 μm and re-simulating the hemodynamic and BOLD responses in sVAN1, we were able to generate an initial dip (dashed gray line in **Supplementary Figure S4**), thus confirming that low baseline blood velocity in capillaries can contribute to a BOLD initial dip.

Next, we tested whether a manipulation of a reconstructed VAN model that originally produced a BOLD initial dip could act to remove this feature of the BOLD response. Specifically, we manipulated rVAN1 by connecting the arterial inlets to a single supplying vessel and all venous outlets to a single draining vessel, and thereby increased the baseline blood velocity. (Intuitively, all inlet arteries will have different blood velocities, some fast and some slow, and by connecting them together, we force all the velocities to be similar, which slows down some vessels but, importantly, it increases the velocities in the slowest vessels.) After simulating the hemodynamic and BOLD responses with this manipulated version of rVAN1, the BOLD initial dip was no longer present (dashed black line in **Supplementary Figure S4**). Thus, we have demonstrated both how we can manipulate an sVAN to generate a BOLD initial dip and how we can also manipulate an rVAN to eliminate the BOLD initial dip.

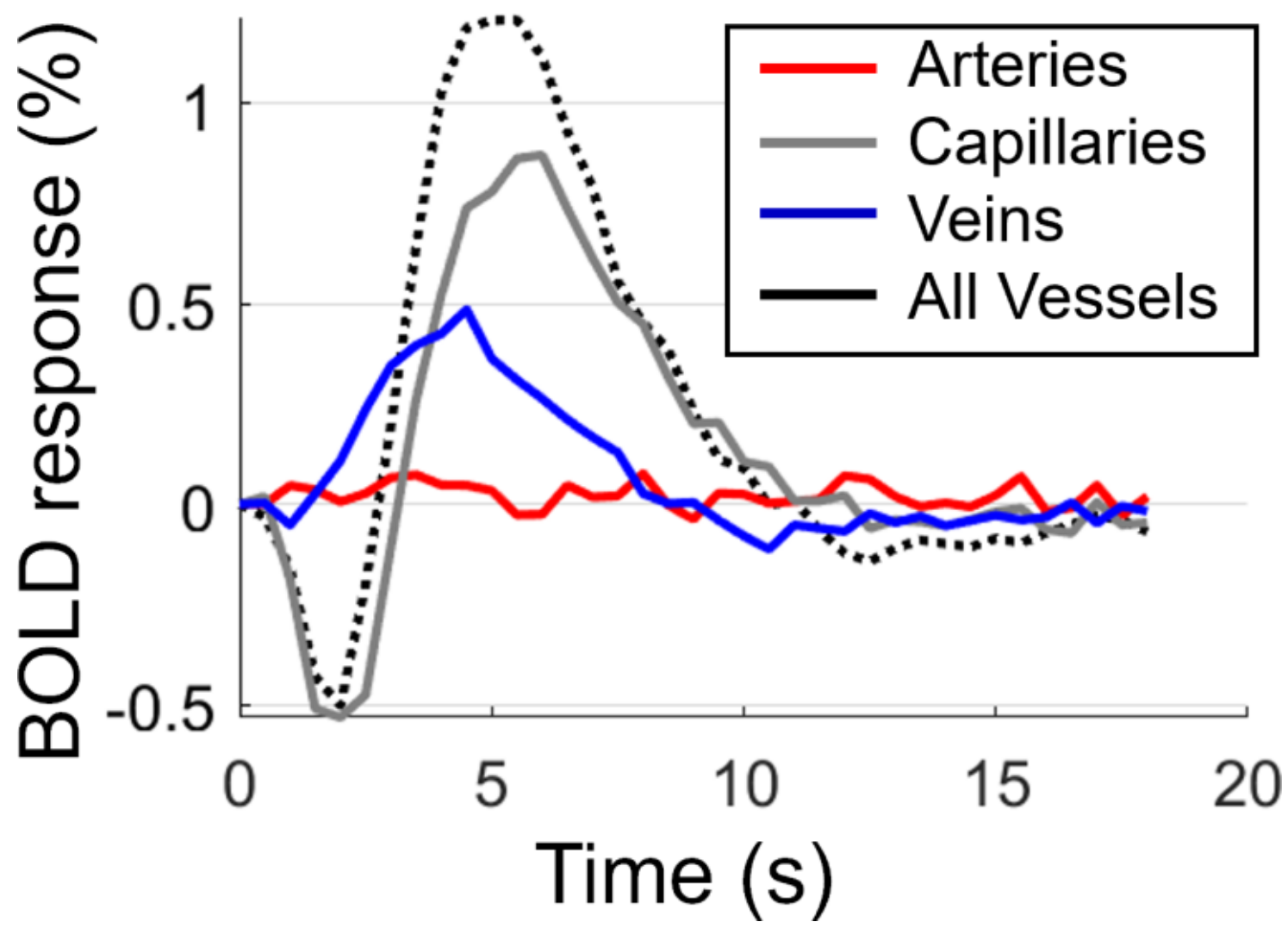

Supplementary Figure S3. Simulated BOLD response of individual vascular compartments. BOLD response time courses from rVAN1, which exhibited a prominent initial dip. Here the BOLD response is shown from individual vascular compartments, the arterial response is shown in the red trace, the capillary response in the black trace, the venous response in the blue trace. The BOLD response is also shown for all vessels combined (gray trace) for comparison. The arterial BOLD response is negligible, whereas the capillary, venous, and all-vessel BOLD responses are apparent. An initial dip is seen primarily in the capillary BOLD response, suggesting that this component is the dominant source of the initial dip in the all-vessel response.

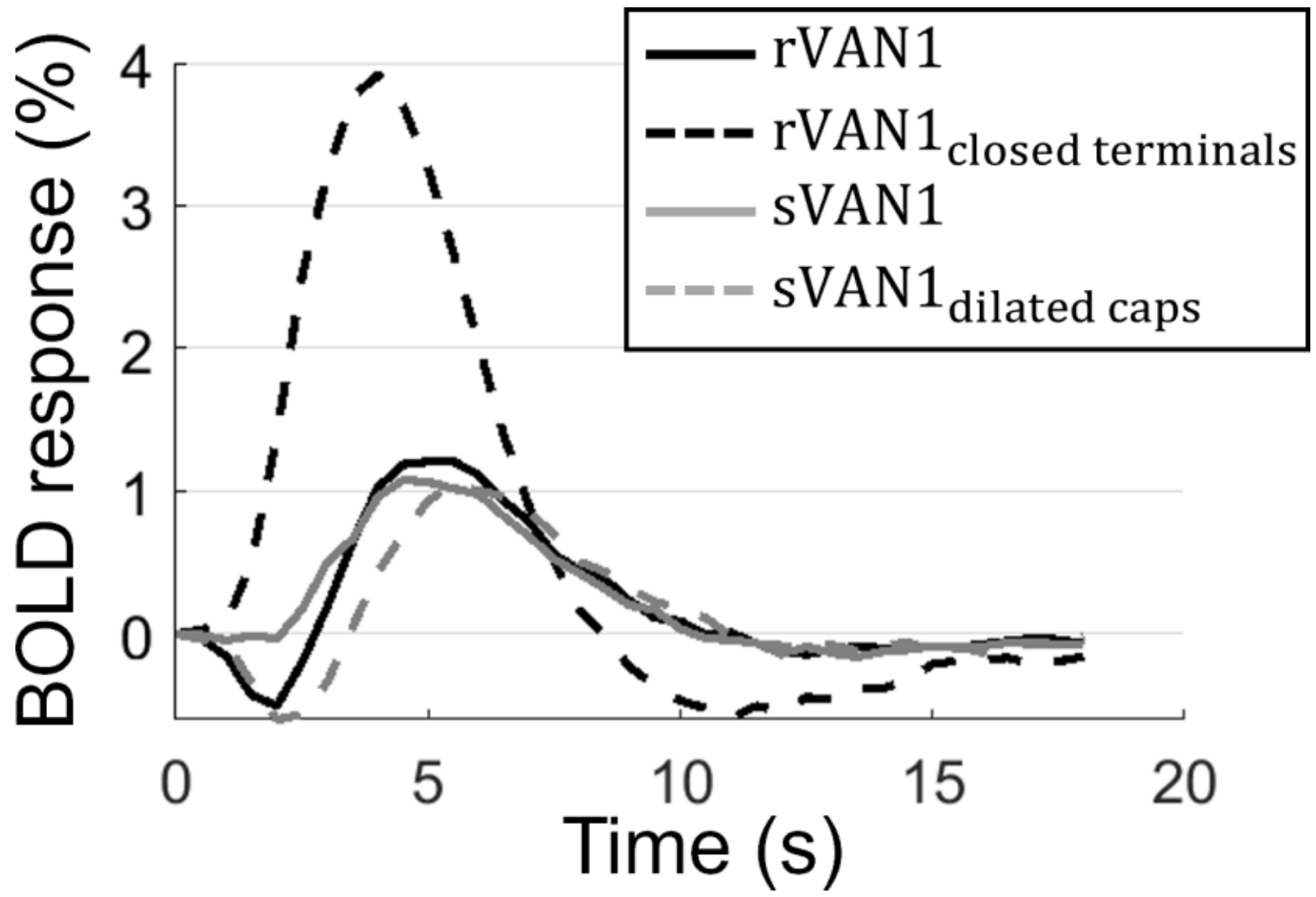

Supplementary Figure S4. Comparison between BOLD response time-courses and the initial dip in a reconstructed and synthetic VAN. The original rVAN1 (solid black line) reflects an initial dip while the original sVAN1 (dashed black line) does not. The initial dip appeared after dilating the capillaries by 2 μm in sVAN1 (dashed gray line). Conversely, by connecting all inlet and outlet terminals to a single inlet and outlet, the initial dip disappeared in rVAN1 (solid gray line).

4. Model sensitivity to input parameter values

Our model requires many input parameter values to be set or derived from empirical data. In order to evaluate the sensitivity of our results to these parameter choices, we varied these input parameters (metabolism, $CMRO_2$, perfusion flow, endothelial permeability, and the diffusivity of oxygen through the parenchyma) and simulated across our validation rVAN (Gagnon et al., 2015) used in the Methods section of the main manuscript. The values used for each parameter are shown in **Supplementary Table S1** and the resulting oxygen and BOLD response profiles are shown in **Supplementary Figure S5**. Expected ranges of input and output values are tabulated in **Supplementary Table S2.** Note, we fixed the lower range of values for perfusion flow at 90 mL/100 g/min as the lower values reduced the flow to the point where the dilations caused substantial flow stalling and, in some cases, unstable calculation of the oxygen tension. Thus, we only investigated the realistic range and omitted cases where the response was unstable due to low flow.

4.1 Baseline oxygen sensitivity

The baseline oxygen tension (pO_2) in tissue and vasculature both diminish strongly with increasing $CMRO_2$ and OEF_{O2}; see **Supplementary Figure S5A**. Note, due to the nonlinear relationship between $CMRO_2$ and OEF_{O2} (See **Supplementary Notes, Section 5**), the relationship between baseline intravascular (IV) and extravascular (EV, tissue) oxygen tension decreases nearly linearly with increasing OEF_{O2}. The tissue oxygen diffusivity, however, has little effect on the baseline pO_2 of either compartment. An increase in perfusion flow, however, increases the amount of oxygen entering the system (as the concentration at the inlet artery is fixed, so an increase in volumetric flow increases the moles of oxygen delivered) and thus increases the intravascular and extravascular oxygen tension. An increase in endothelial permeability increases the EV oxygen tension while decreasing the IV oxygen tension as more oxygen is leaving the blood and entering the tissue.

4.2 Dynamic blood oxygen partial pressure changes, pO_2

The intravascular oxygen deviation is characterized here by the relative oxygen content of each vessel at the time of peak arterial oxygen content. This deviation increases as a function of increased $CMRO_2$ and OEF_{O2}, caused by the low baseline oxygen content. Again, the nonlinear relationship between $CMRO_2$ and OEF_{O2} causes a nearly linear relationship between OEF_{O2} and oxygen deviation; see **Supplementary Figure S5B**. The oxygen deviation remains highly stable across a range of diffusivity values. An increase in perfusion flow led to a marked decrease in oxygen deviation from baseline, ostensibly due to higher oxygenation of vessels at baseline which decreases the potential for deviation during activation. Increasing the endothelial permeability led to an increase in oxygen deviation, again due to a decrease in baseline vascular oxygenation and increasing the capacity for change during activation. We do note, however, that the endothelial permeability remains relatively constant near the ranges used in this study.

4.3 BOLD

The BOLD response amplitude (taken as the peak of the positive BOLD response) increased as a function $CMRO_2$ and OEF_{O2} values until the highest values of $CMRO_2$ and OEF_{O2} were reached,

at which point it decreased to a value of 0. The general trend of increasing BOLD response comes from a marked decrease in baseline oxygenation and increase in oxygen change, but at the extrema, the metabolism was sufficiently strong that the flow response could not compensate and create a net decrease in deoxyhemoglobin concentration. As with baseline oxygen and oxygen deviation, the BOLD response amplitude remains stable across all tissue oxygen diffusivity values tested. Increases in perfusion flow led to a decrease in the BOLD response magnitude which follows the oxygen response curve. The endothelial permeability also follows the oxygen response curve, increasing the BOLD response amplitude with larger permeability values.

Supplementary Table S1. Value ranges for sensitivity analysis.

Property	Values	Units
Metabolic rate ($CMRO_2$)*	[0.0001, 0.001, 0.01, **0.02526**, 0.1, 1, 10]	s^{-1}
OEF*	**calculated from $CMRO_2$	%
Perfusion flow	[90, **100**, 110, 125, 150, 175, 200, 500, 1000]	mL/100g/min
Parenchymal diffusivity	[0.1, 1, 10, 100, 1000, **1800**, 10000, 100000]	$\mu m^3/s$
Endothelial permeability	[0.1, 1, 10, 100, 1000, **2400**, 10000, 100000]	$\mu m^3/s$

*$CMRO_2$ and OEF are scaled forms of the same measurement

Supplementary Table S2. Expected ranges of several physiological parameter values from mouse used in the VAN modeling framework and for our sensitivity analysis.

Parameter	Expected values	Units	References
$CMRO_2$*	0.25–2.96	μmol/ml/min	(Fang et al., 2008; Gagnon et al., 2015; Moeini et al., 2018; Sakadžić et al., 2016; Zhu et al., 2002)
Perfusion flow	40–170	ml/100 g/min	(Gertz et al., 2006; Maeda et al., 2000; Xu et al., 2011)
Baseline pO_2	26.1–142	mmHg	(Vazquez et al., 2010)
Intravascular pO_2 change	0.7–16.4	mmHg	(Vazquez et al., 2010)
BOLD response (to a 2-second stimulus)	1–2	%	(Bernard et al., 2023; Desjardins et al., 2019)

*$CMRO_2$ values were estimated assuming a mean pO_2 of 35 mmHg for the healthy adult mouse (Moeini et al., 2018).

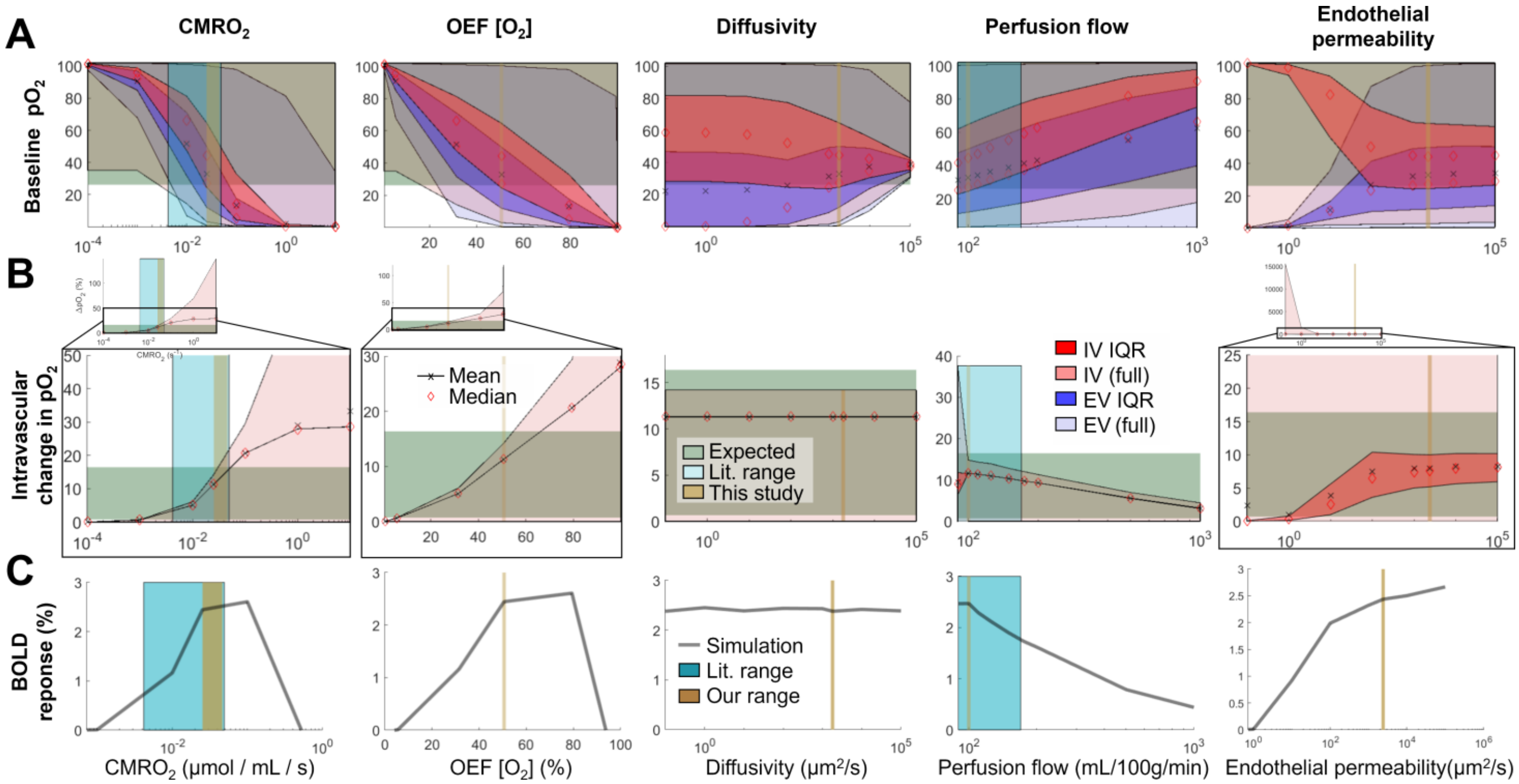

Supplementary Figure S5. Sensitivity to input parameter values reflects that when using experimentally derived values, the resulting simulation outputs fall within reported values. In all cases, the range of values in our simulations (orange region) yields results within the expected values (from literature, green region). Note, our value ranges also lie within the literature range (yellow region). The baseline oxygen (A), oxygen deviation (B), and BOLD responses (C) all show that the oxygen tension remains stable over a large range of input parameters. Variation in oxygen diffusivity shows the least impact on the simulations, as exemplified by the intravascular (IV, red) and extravascular (EV) compartments in both the interquartile range (IQR) and the full range of data (FR). An increase in the metabolic rate ($CMRO_2$ and OEF) causes a decrease in baseline pO_2 and an increase in pO_2 response which translates, generally, to an increase in the BOLD response. However, after a "tipping point", such a large metabolic rate results in a negligible BOLD response. In contrast, an increase in perfusion flow translates to an increase in baseline pO_2 but a decrease in pO_2 response and BOLD response. A decrease in vessel wall permeability decreases the EV pO_2 while increasing the IV pO_2. While increasing permeability, the two converge to similar pO_2 values.

5. Nonlinear relationship between OEF and $CMRO_2$

We note that in our simulations $CMRO_2$ is computed within each mesh element, and is defined as the amount of oxygen consumed per unit volume. OEF, however, is calculated as the difference in oxygen content between the large feeding arteries and large draining veins (inlets and outlets of our simulation domain) at the scale of the entire fMRI voxel containing the VAN model. However, the oxygen tension varies spatially throughout the voxel in our simulations (e.g., higher oxygen tension near feeding arteries). Thus, any changes in the vascular geometry (e.g., vessel position, connectivity, vessel wall surface area, etc.) will lead to changes in OEF values at the voxel-level for the same $CMRO_2$ value. This spatial variability in oxygen content leads to an observed nonlinearity between OEF and $CMRO_2$ as observed in **Supplementary Figure S6.**

We also note that Michaelis-Menten kinetics may also be considered, however this would only be valid for a diffusion-limited system, which is not the case for oxygen in the human or mouse brain.

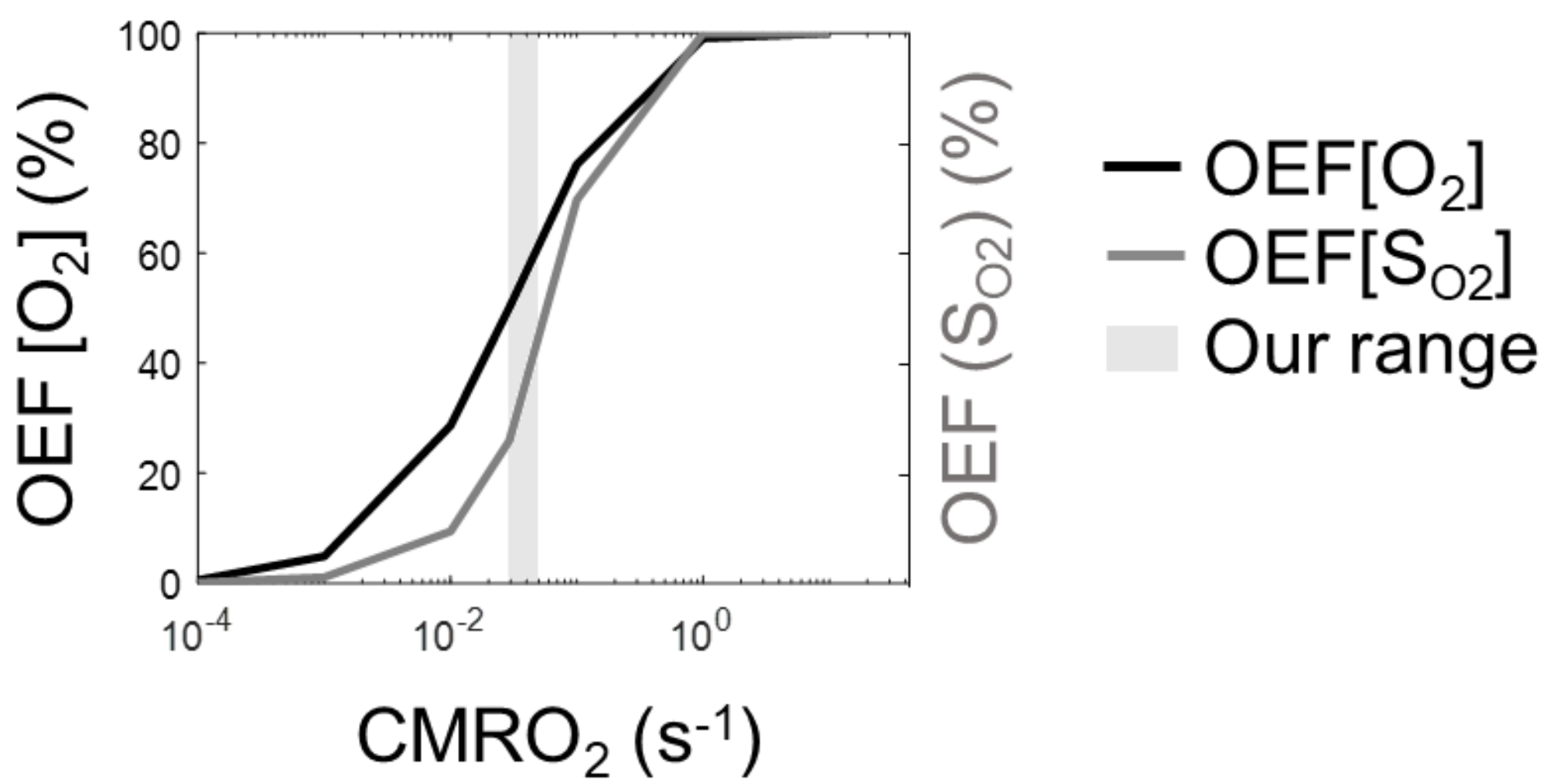

Supplementary Figure S6. Visualization of the relationship between input parameter $CMRO_2$ and different computations of the oxygen extraction fraction (OEF). The OEF [O_2] (left axis, black line) represents the loss of oxygen directly while OEF (S_{O2}) (right axis, gray line) reflects the OEF computed conventionally from oxygen saturation.

6. MAB principle observed in reconstructed VAN models

As noted in the main text, the MAB principle was observed in all reconstructed VANs. Examples from two VAN datasets measured from two different laboratories (Blinder et al., 2013; Gagnon et al., 2015) are shown in **Supplementary Figure S7**. These VANs exhibit an asymmetry in diving-arterial and ascending-venule branches and the MAB property.

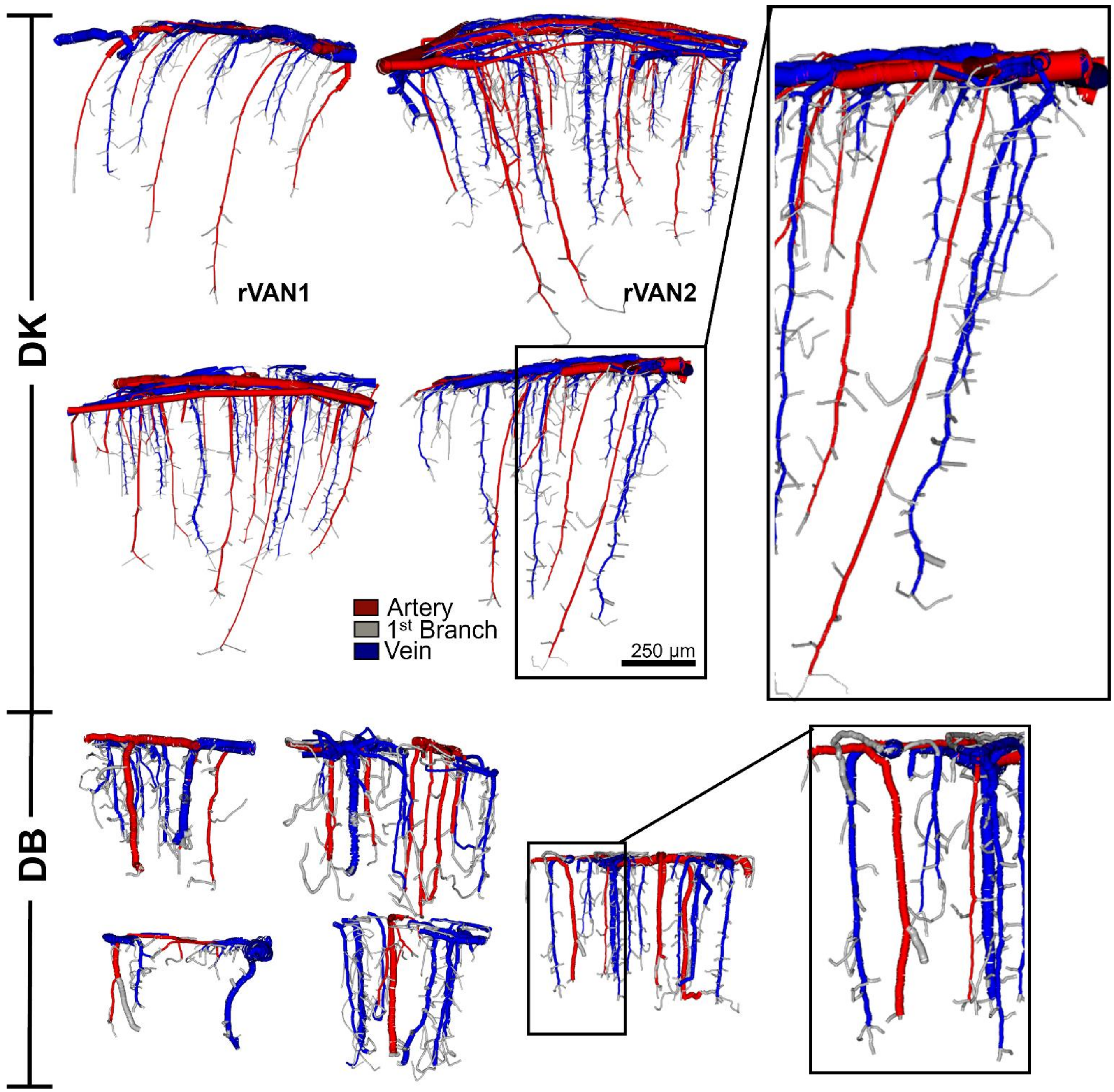

Supplementary Figure S7. Visualizations of arteries, veins and first branches in two sets of reconstructed VANs (Blinder et al., 2013; Gagnon et al., 2015). The number and density of branches on the diving arteries is lower than those on the ascending veins. The "DK" VANs are those provided by the David Kleinfeld laboratory, and the "DB" VANs are those provided by the David Boas laboratory.

7. VAN synthesis algorithm updates

The geometric and topological inputs to the synthesis methodology are given in **Supplementary Table S3.** We modified the original iCNS algorithm as explained in the *Online Methods* of the main text. One of the key updates was a change to how tortuosity is imposed on the vessel segments at the final stage of the synthesis. The tortuosity (T) is of a curved segment is defined as the full pathlength of the segment divided by the Euclidean distance between the start and end points of the segment, such that tortuosity is ≥ 1, and is computed as:

$$T = \frac{\sum_{i=1}^{n} |P_i - P_{i-1}|}{|P_n - P_0|} \tag{2}$$

where the segment consists of *n+1* nodes and *n* edges, $|P_n - P_0|$ indicates the Euclidean distance between the segment terminals (the branching points that start and end the segment), and $|P_i - P_{i-1}|$ is the length of edge i. In the final synthesis stage, the geometric properties of diameter, tortuosity, and length are matched by replacing each synthetic straight vessel segment with a more anatomically realistic vessel segment that exhibits curvature and tortuosity. Our new method replaces the straight vessel segments with reconstructed vessel segments taken from a dictionary derived from anatomical data. The vessel dictionary used here was generated directly from the vessels from one of the rVANs used for simulations (Gagnon et al., 2015). The replacement method simply identifies a dictionary entry (i.e., a real vessel segment from the anatomical data) for each synthesized straight segment based on the desired tortuosity. The desired tortuosity is calculated as described previously (Hartung, Badr, Mihelic, et al., 2021). This vessel will then be rotated, scaled, and translated into the place of the original, synthesized straight segment. Once in place, if this new vessel from the dictionary intersects any of the nearby vessels in the VAN, the new entry is rotated until it no longer intersects with any other vessel.

The details of the rotation, scale, and placement of the tortuous vessels are given here in sufficient detail for re-implementation. The procedure takes a vessel from the dictionary, translates and scales it into the correct position before rotating it until it no longer intersects any other vessels. The procedure for replacing the original straight vessel segment with a vessel segment from the dictionary starts by defining unit vectors created from the endpoints of the segments as:

$$u = \frac{(P_n - P_0)}{|P_n - P_0|}, \qquad v = \frac{(P'_n - P'_0)}{|P'_n - P'_0|} \tag{3}$$

where the normalized vectors u and v are formed from the start and end points of the original straight segment and the tortuous candidate segment as shown in **Supplementary Figure S8**. We can then define a vector perpendicular to both vectors (a):

$$a = v \times u \tag{4}$$

This vector (a) will be the axis over which the new tortuous vessel will be rotated, if needed. This can be used to define a new intermediate matrix (A):

$$A = \begin{bmatrix} 0 & -a_z & a_y \\ a_z & 0 & -a_x \\ -a_y & a_x & 0 \end{bmatrix} \tag{5}$$

where <a_x, a_y, a_z> are the x, y, and z elements of the a vector. This matrix can now be used to create the rotation matrix (R):

$$R = I + A + A^2 \frac{1 - v \cdot u^T}{|v|^2} \tag{6}$$

And the scaling (s) can be defined as:

$$s = \frac{|P_n - P_0|}{|P'_n - P'_0|} \tag{7}$$

Therefore, the rotation, scaling, and translation can be defined for all points in the tortuous segment (P'_i) to become ($P_i^{\prime,\text{new}}$) by the operation:

$$P_i^{\prime,\text{new}} = R(P'_i - P'_0)s + P_0 \tag{8}$$

Then, to rotate the new tortuous vessel along its axis to avoid intersecting other vessels, a similar operation can be made by using the new intermediate matrix (W):

$$W = \begin{bmatrix} 0 & -u_z & u_y \\ u_z & 0 & -u_x \\ -u_y & u_x & 0 \end{bmatrix} \tag{9}$$

This matrix can now be used to create the new rotation matrix (Q):

$$Q = I + W \sin(\theta) + W^2(1 - \cos(\theta)) \tag{10}$$

where θ is the rotational angle about the axis formed between the endpoints of the original segment. In our algorithm, a process iterates through θ until a satisfactory orientation is achieved. In the event a segment cannot be placed without exiting the VAN bounds or intersecting another segment, it is flipped and the process is repeated.

Supplementary Table S3. Input parameters and values used in vascular synthesis for mouse and human VANs.

	Property	Mouse Value	Human Value
**G**	Diving arterial density [mm^{-2} cortical surface]	9.0	2.0
	Ascending venule density [mm^{-2} cortical surface]	27.0	1.0
	Capillary density [mm^{-3} parenchyma]	11,600	8,800
	Cortical thickness [mm]	1.1	2.5
**T**	Ratio of diving artery to ascending venule	3.0	0.5
	Arteriolar first branches* [mm^{-1}]	5.0	4.3
	Venular first branches* [mm^{-1}]	12.5	13.1

G: geometric properties, **T**: topological properties.
*: First branching vessels are parameterized as branches per length of penetrating vessel

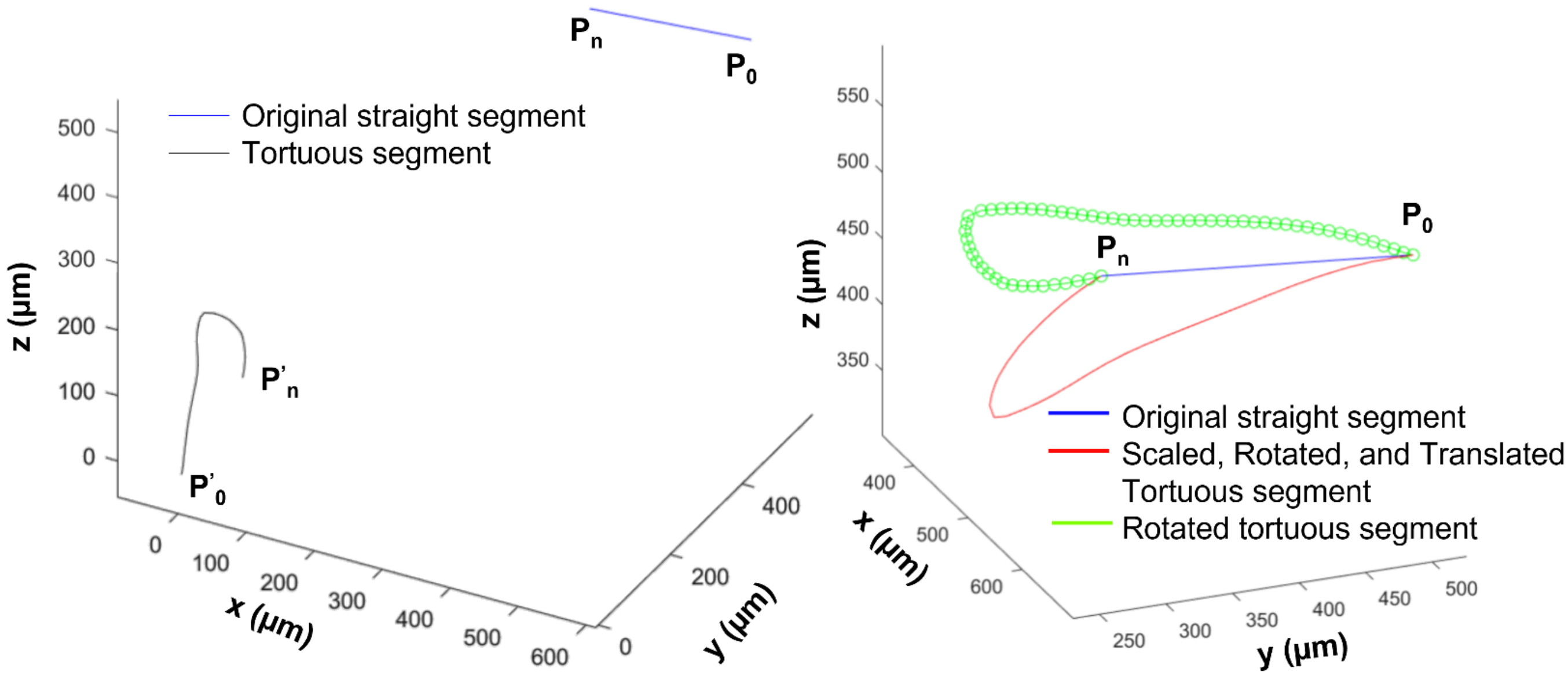

Supplementary Figure S8. Method of incorporating tortuosity into synthetic VANs. Here we present an example of a straight segment generated during synthesis (blue line) and its replacement tortuous segment (black line). Points P_0 and P_n represent the coordinates of are the endpoints of the original straight vessel segment, and points P_0' and P_n' represent the endpoints of the chosen tortuous vessel segment from the dictionary. (Left) The original and tortuous segments do not match in length or position. (Right) After rotation, alignment, and translation the tortuous segment matches the position and endpoints of the original segment. Also shown here is a rotated version of the original segment (green line with circular markers indicating the internal nodes of the segment).

8. Vascular relabeling for assigning arterial dilations

Because the arterial dilation recordings are indexed by cortical depth and by branching order, it is important for the VAN models to accurately assign the diving arterial vessels and the correct branching order to each active segment. Previous labeling provided with reconstructed VAN datasets contain several instances where branching arterioles and diving arterioles are labeled in the same compartment when they should be consistently assigned to different compartments so that the application of our dilation traces is valid. It is important to note that this labeling choice that we define here is not necessary for all other analyses or simulations, but in our particular application is important for the anatomical labeling to be consistent with the definitions used in the reporting of recorded active dilation traces. Therefore, to ensure appropriate assignment of our dilation traces to diving arteries and branching arterioles, we automatically analyzed the branching orders and relabeled vessels to separate diving arteries from all other branching arterioles and capillaries. Our relabeling resulted in many vessels originally labeled as arterioles to be relabeled as diving arteries and many others to be relabeled as capillaries (to be used as branching arterioles). In some cases, we also relabeled the veins using the same algorithm, although it had no impact on the hemodynamic response because the ascending veins and capillaries observed the same passive dilation mechanics.

Here we describe our algorithm from the perspective of cleaning arterial trees, although the same algorithm can be applied to veins. Our automated algorithm began with the originally labeled diving arteries. At any bifurcation where both child branches were labeled as diving arteries, at least one of the branches was considered an incorrect label. When this happens, one of the child branches is considered the “true” diving artery while the other is considered mislabeled. The “true” diving artery was determined by a winner-take-all criterion that finds the most vertical segment. Verticality was defined by an axis along the VAN, essentially the shortest path from the white-matter interface to the CSF interface. The smallest angular difference between the segment and this axis was chosen to be the most vertical and the other branch was relabeled as a capillary. All downstream arteries from this vessel were also relabeled as capillaries.

In some cases, neither child branch was considered “vertical” and both child branches were considered capillaries. This was identified when the winner at a bifurcation was at an angle >45°. We show in **Supplementary Figure S9** that this algorithm removed arterioles from the diving arteries and venules from the ascending veins. **Supplementary Figure S10** shows the effect of relabeling on another reconstructed VAN, rVAN2.

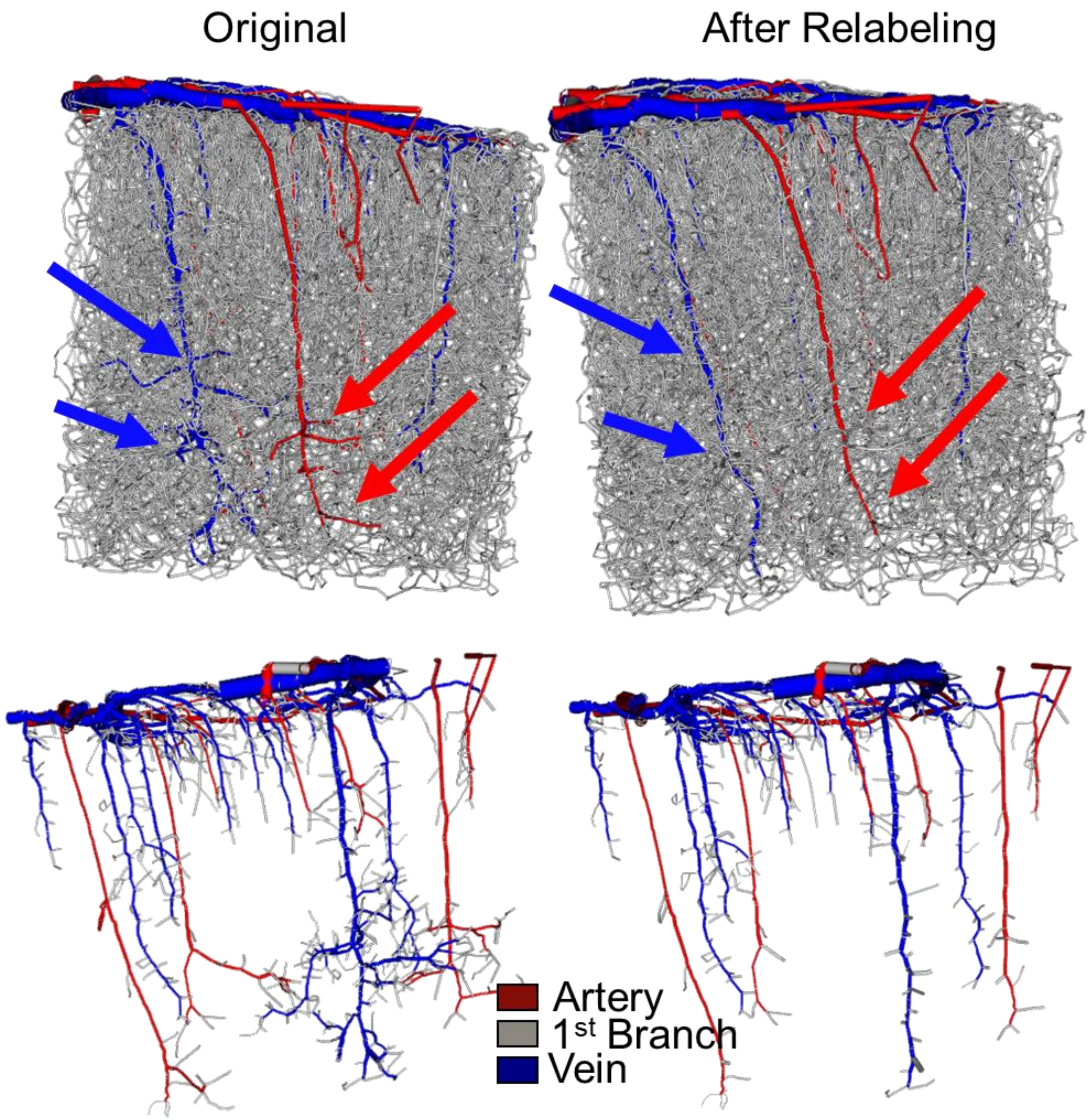

Supplementary Figure S9. Correction of vessel labels in reconstructed VAN models. Three-dimensional rendering of rVAN1 before and after relabeling with some examples of relabeled vessels as annotated with arrows in red representing diving arteries and blue representing ascending veins. Visualizations verify that diving arteries and ascending veins are preserved while branches are relabeled in the capillary group. The structure remains overall unchanged (no vessels are removed, only relabeled).

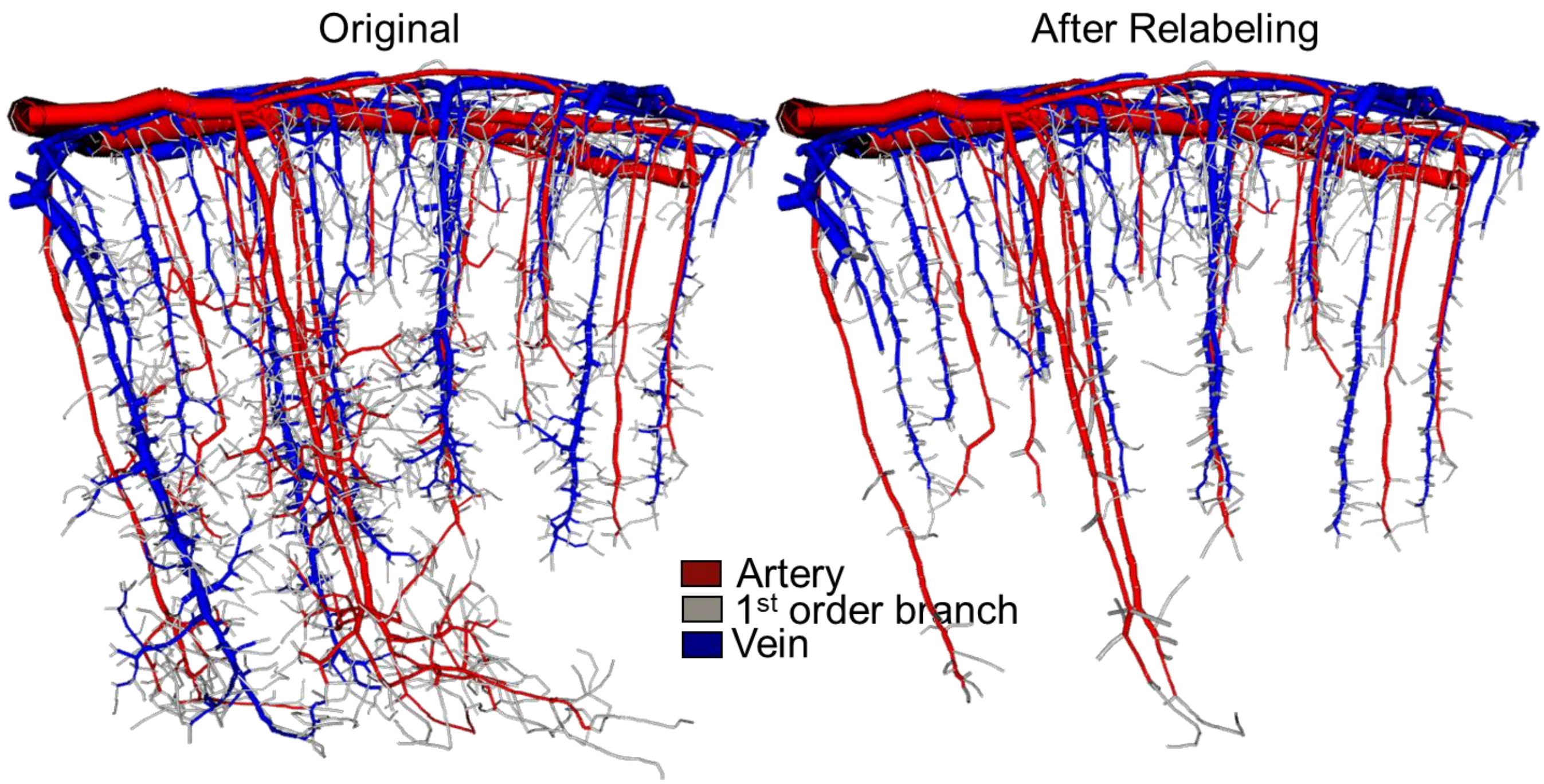

Supplementary Figure S10. Additional example of vessel relabeling. Three-dimensional rendering of rVAN2 before and after relabeling the branches from the diving arteries and ascending veins. Here arteries are shown in red, veins are shown in blue, and first branches are shown in gray. After relabeling, many branches in the original dataset are reclassified as capillaries.

9. Hemoglobin binding kinetics

Oxygen is transported through the bloodstream by two mechanisms. The first mechanism is the oxygen advected through the plasma surrounding the red blood cells (RBCs). The second mechanism is oxygen bound to one of the four binding sites on hemoglobin molecules within RBCs. The oxygen can bind and un-bind from the hemoglobin as described previously based on how much free oxygen is in the plasma surrounding the RBCs. At steady state, the amount of oxygen attached to RBCs, denoted as the saturation of hemoglobin, has been characterized by the well-known Hill equation (Hill, 1910)

$$S = \frac{1}{1+\left(\frac{p_{50}}{pO_2}\right)^c}, \tag{11}$$

where S is the saturation of hemoglobin, c is the Hill constant, p_{50} is the partial pressure at 50% saturation and pO_2 is the current partial pressure of oxygen. This equation relates the free oxygen to the saturation of hemoglobin as a function of the partial pressure of oxygen in the plasma. The hemoglobin concentration can then be estimated by the RBC volume and the hemoglobin density per RBC:

$$V_{\mathrm{Hb}} = \frac{V_{\mathrm{v}}\, H\, d_{\mathrm{Hb}}^{\mathrm{RBC}}}{V_{\mathrm{RBC}}} \tag{12}$$

where V_{v} is the volume of the blood vessel in question, V_{RBC} is the volume of a single RBC, V_{Hb} is the volume of hemoglobin, H is the vessel hematocrit, and $d_{\mathrm{Hb}}^{\mathrm{RBC}}$ is the density of hemoglobin in each RBC. The Hill equation, though, describes the steady-state saturation yet this exchange between free oxygen in plasma and bound oxygen on hemoglobin is dynamic. We thus approximate the reversible hemoglobin-oxygen binding system with a 1st-order reaction sequence:

$$Hb^{\mathrm{O}} \underset{k^-}{\overset{k^+}{\leftrightarrow}} O + Hb$$

where Hb^{O} is hemoglobin-bound oxygen, Hb is deoxyhemoglobin, O is free oxygen in the plasma, k^+ is the kinetic rate for the forward reaction (dissociation), and k^- is the kinetic rate of the reverse reaction (binding). The ratio of these kinetic rates (k^+/k^-) can be fit to the Hill equation as shown in **Supplementary Figure S11**. The linear reaction rate was approximated by fitting to the value of the Hill equation at a pO_2 value of 78.74 mmHg (as indicated in **Supplementary Figure S11**) resulting in a reaction rate ratio (k^+/k^-) of 18.75 s^{-1}. The precise value of the kinetic rates (k^+ and k^-) were then chosen to fit this ratio and match the steady-state oxygen from previously simulations (Gagnon et al., 2015).

Once the kinetic rates are chosen, the kinetic equations for binding and unbinding can be written as:

$$\frac{\partial n_{\mathrm{v}}^{\mathrm{Hb}}}{\partial t} = k_{\mathrm{Hb}}^{-} n_{\mathrm{v}}^{\mathrm{P}} - k_{\mathrm{Hb}}^{+} n_{\mathrm{v}}^{\mathrm{Hb}} \tag{13}$$

$$\frac{\partial n_{\mathrm{v}}^{\mathrm{P}}}{\partial t} = k_{\mathrm{Hb}}^{+} n_{\mathrm{v}}^{\mathrm{Hb}} - k_{\mathrm{Hb}}^{-} n_{\mathrm{v}}^{\mathrm{P}} \tag{14}$$

where k_{Hb}^{+} is the linear reaction rate of the dissociation, k_{Hb}^{-} is the reaction rate of binding, $n_{\mathrm{v}}^{\mathrm{P}}$ is the number of free oxygen molecules in the plasma, and $n_{\mathrm{v}}^{\mathrm{Hb}}$ is the number of molecules of oxygen bound to hemoglobin.

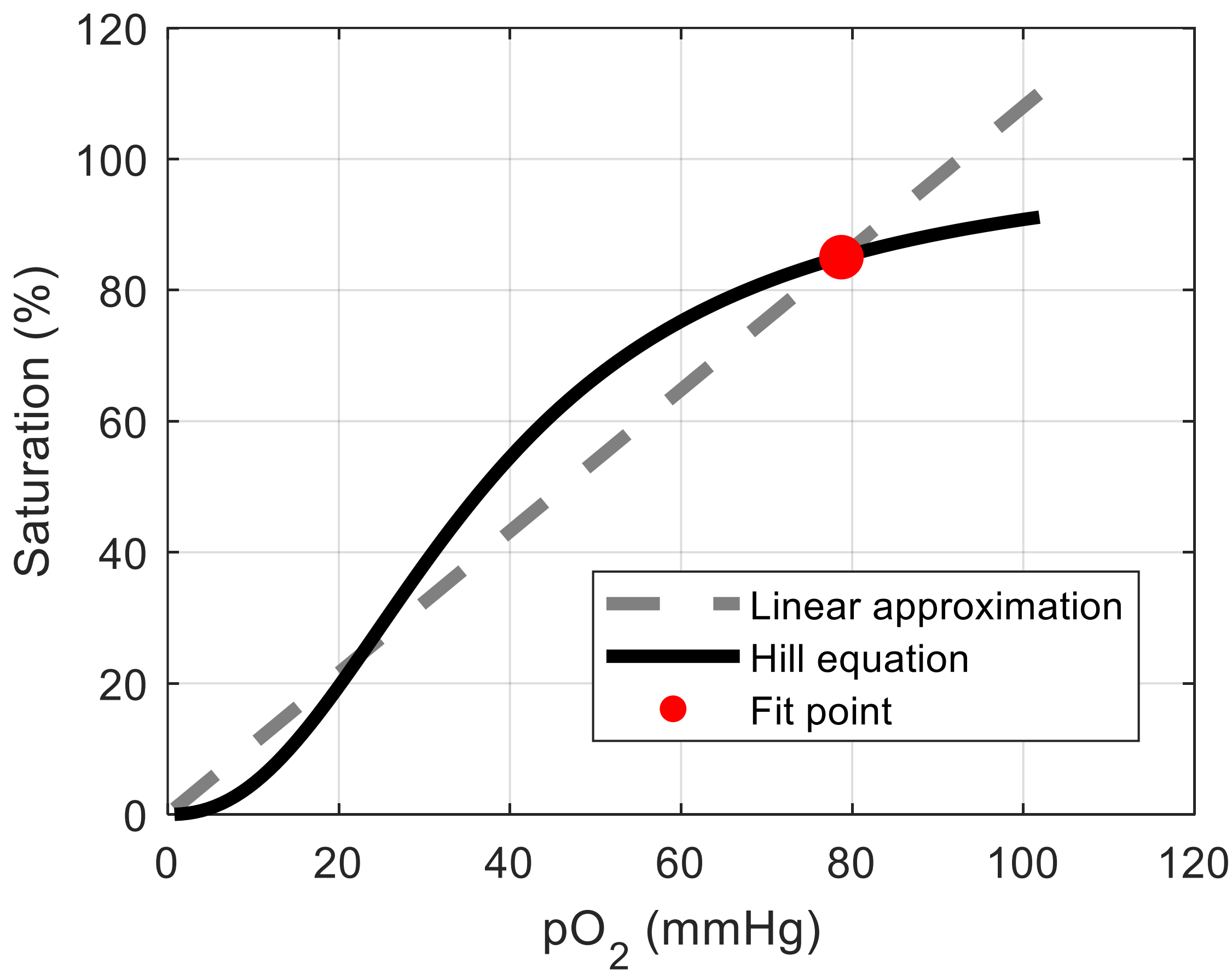

Supplementary Figure S11. Linearization of the Hill equation. The linear approximation (gray dashed line) of the hemoglobin-binding kinetics of oxygen used in our simulations compared to the nonlinear Hill equation (solid black line). We report here the saturation of hemoglobin as a function of partial pressure of oxygen. The fit point of the linearized model is marked by a red circle.

10. Parameter fitting for arterial traces in human cortex

As described in the *Methods* of the main text, we used measurements of single-vessel arterial dilations from rodents to approximate dilations in humans. Because the recorded dilations are provided as a function of cortical depth in the mouse cortex, it was necessary to both interpolate and extrapolate the values to adapt the recordings to the human VAN that spans a much thicker human cerebral cortex; an example extrapolation was presented in **Figure 2B** in the main text. These extrapolations from the mouse to the human cortex were generated through simple regression of cortical depth across the four parameters representing the temporal features of the dilation traces that most clearly vary with depth in the rodent data (onset time, amplitude, time-to-peak, and time-to-undershoot). Note that, as described in the *Methods*, we used a simple linear fit for three out of the four model parameters since these all appeared to vary linearly with cortical depth. However, because the peak amplitude did not appear to vary linearly with depth, we fit this instead with the next simplest model: a second-order polynomial. These first- and second-order polynomial fits, which provided the bases for the extrapolations, are presented in **Supplementary Figure S12**.

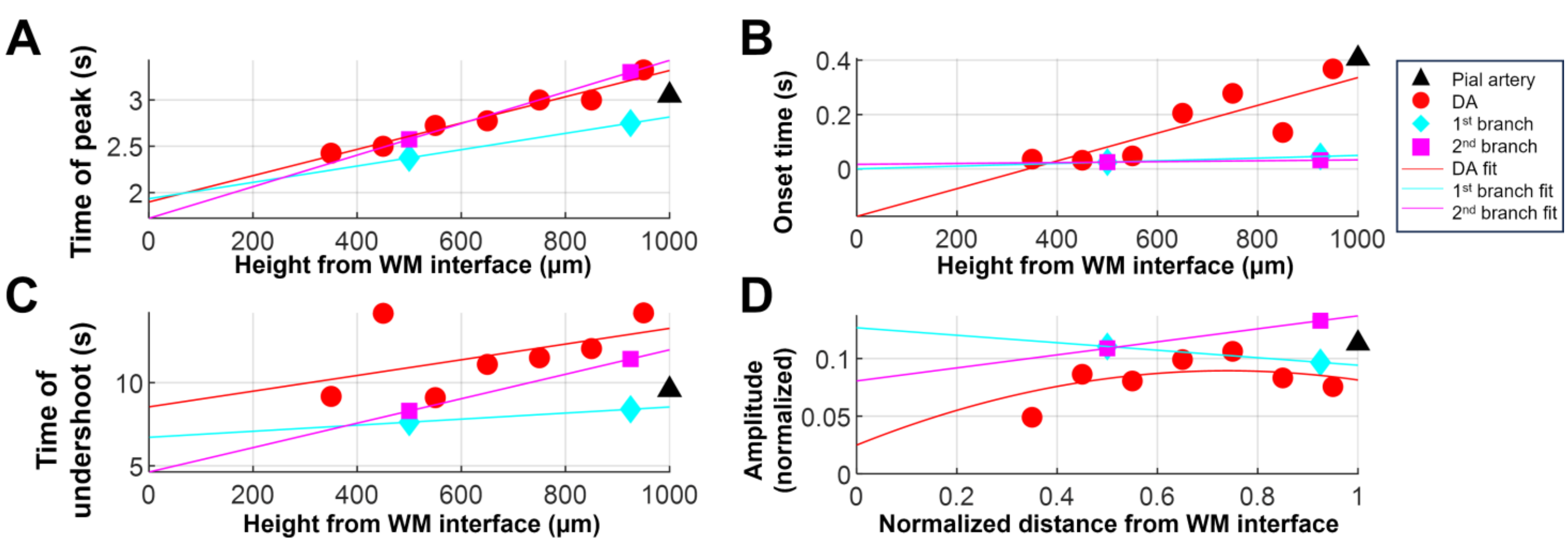

Supplementary Figure S12. Fitting the four parameters of the mouse data with polynomial regression. (A) We extrapolated four parameters, (A) peak dilation time, (B) onset time, and (C) time of the posts-stimulus undershoot, using a first-order polynomial (i.e., a straight line). Note, panel (B) is the same as shown in **Figure 2** of the main text but without the extrapolated section. (D) For the peak amplitude we chose a second-order polynomial for regression. Note that the peak amplitude has been shown to vary systematically with relative cortical depth, i.e., the cortical depth normalized by the cortical thickness, so the peak amplitude will occur roughly within the same cortical layer in the human and mouse cortex.

11. Varying arteriolar branching in reconstructed VAN 1 to test MAB principle

In order to directly assess the impact of the relative numbers of branching arterioles and branching venules on the BOLD response in a way that is analogous to our resistor model simulations presented in **Figs. 4D** & **E**, we devised a new approach to "edit" the rVAN1 model to add arterioles and remove venules, creating two new, edited versions of rVAN1; (i) rVAN1 with symmetric branching and (ii) rVAN1 with more branching arterioles than branching venules that we refer to as "reverse MAB". To generate an edited rVAN model with symmetric branching, we added new arterioles between existing nodes of the penetrating arterial tree and connected them to the middle of the nearest capillary segment to create new arteriolar branches until the number of arterioles and venules match, creating the rVAN1 with symmetric branching. To generate an edited rVAN model with reverse MAB, we removed branching venules until the number of branching venules matched the number of branching arterioles in the original rVAN1. (We were careful not to remove any branching venules that would create new terminals in the vascular structure.) These steps are visualized in **Supplementary Figure S13**.

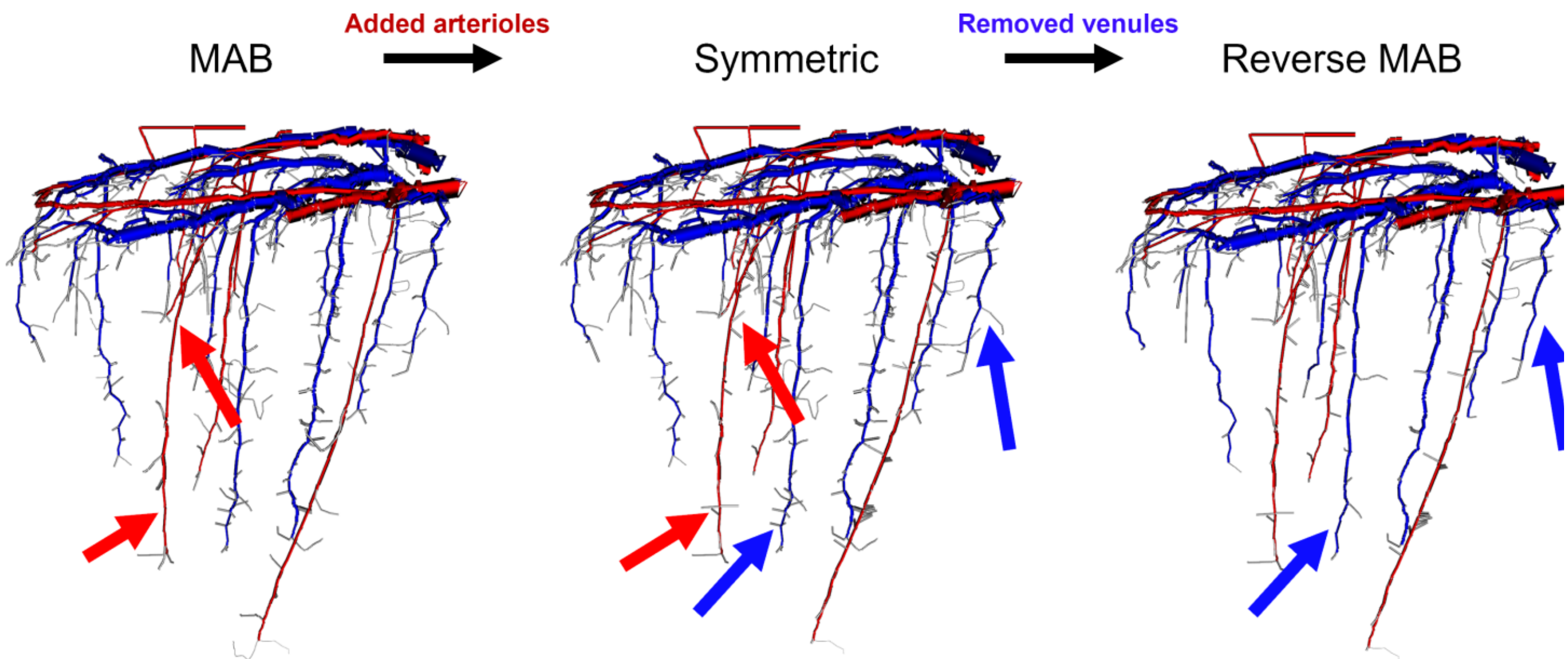

Supplementary Figure S13. Visualization of the edits to rVAN1 that created symmetric arteriolar branching and reverse MAB versions of a reconstructed VAN model. From left to right, visualization of the branches (in gray) from the original rVAN1 reflect more branches on intracortical veins than on arteries (i.e., the MAB principle). To generate an edited rVAN model with symmetric branching, we added arterioles until the number of arteriolar branches and venular branches were equal. To generate an edited rVAN model with reversed MAB, we then removed venular branches until the number of branching venules was equal to the number of branching arteries in the original rVAN1.

Supplementary Table S4: List of simulation constants, along with cited referencess (Refs.) from which the value was taken. Parameter values relevant for our simulations that do not explicitly appear in any equations in the main text or *Supplementary Notes*, but that are used in our software and thus relevant to reproduce our results, are indicated with an asterisk (*).

Name	Symbol	Value	Units	Refs.
Vessel compliance parameter	β	1	–	(Boas et al., 2008; Gagnon et al., 2015)
Vessel elasticity and tissue viscosity parameter	τ	1	–	(Boas et al., 2008; Gagnon et al., 2015)
Long-duration vessel elasticity and tissue viscosity parameter**	τ	20	–	(Boas et al., 2008; Gagnon et al., 2015)
Mass transfer coefficient**	U	2.4×10^3	μm²/s	(Hartung, Badr, Moeini, et al., 2021; Truskey et al., 2004)
Endothelial wall thickness	w	1	μm	(Gagnon et al., 2015; Gould et al., 2017; Hartung, Badr, Moeini, et al., 2021)
Diffusivity of oxygen in tissue	D	1.8×10^3	μm²/s	(Hartung, Badr, Moeini, et al., 2021; Mintun et al., 2001)
Arterial boundary pressure	P_{art}	120	mmHg	(Gould et al., 2017; Hartung, Badr, Mihelic, et al., 2021; Hartung, Badr, Moeini, et al., 2021; A. Linninger et al., 2019; A. A. Linninger et al., 2013)
Venous boundary pressure	P_{vein}	5	mmHg	(Gould et al., 2017; Hartung, Badr, Mihelic, et al., 2021; Hartung, Badr, Moeini, et al., 2021; A. Linninger et al., 2019; A. A. Linninger et al., 2013)
Arterial inlet plasma oxygen concentration	$c_{\mathrm{v}}^{\mathrm{p}}$	102	mmHg	(Gagnon et al., 2015)
Arterial inlet oxygen saturation	SaO_2	92	%	-
Kinetic hemoglobin dissociation rate	k_{hb}^{+}	5.0×10^3	1/ms	-
Kinetic hemoglobin binding rate	k_{hb}^{-}	93,763	1/ms	-
Hematocrit	H	0.1–0.4	–	(Gagnon et al., 2015; Griffeth & Buxton, 2011)
Gyromagnetic ratio of water	γ	2.675×10^2	rad/T/s	(Gagnon et al., 2015)
T_2^* relaxation time of gray matter	$T^*_{2,\mathrm{tiss}}$	50	ms	(Uludağ et al., 2009)
Number of protons in voxel	N	10^7	protons	(Gagnon et al., 2015)
Echo time	TE	30	ms	(Gagnon et al., 2015)
Flip angle	–	90	degrees	(Gagnon et al., 2015)
MRI magnetic field	B_0	7	Tesla	(Gagnon et al., 2015)
Frequency-encoding gradient field	G_{x}	50	mT/m	-
Diffusivity of water in tissue**	$D_{\mathrm{H_2O}}$	10^3	μm²/s	(Gagnon et al., 2015; Pathak et al., 2008)
Susceptibility difference between fully oxygenated and fully deoxygenated blood	$\chi_{\max}$	3.32×10^{-6}	–	(Christen et al., 2012; Gagnon et al., 2015)
Bunsen solubility coefficient of oxygen*	B	1.27×10^3	mmHg μm³/amol	(Gagnon et al., 2015)
Partial pressure of oxygen at 50% saturation**	P_{50}	40.2	mmHg	(Gagnon et al., 2015; Uchida et al., 1998)
Hill constant**	c	2.59	–	(Gagnon et al., 2015; Uchida et al., 1998)
Cerebral metabolic rate of oxygen consumption	k	0.4–0.8	1/s	-

Mouse 0th-order oxygen metabolism rate at baseline*	K_0^{mouse}	1.5–2.8	μmol/ml/min	-
Human 0th-order oxygen metabolism rate at baseline*	K_0^{human}	1.98	μmol/ml/min	-
Oxygen extraction fraction	OEF	30	% by saturation	(Sakadžić et al., 2014)
Cerebral blood flow	CBF	100	ml/100g/min	(Buck et al., 2018; Le et al., 2024; Lee et al., 2022; Munting et al., 2021)

**Estimated from simulations.

10. References

Bernard, R., Valverde Salzmann, M., Scheffler, K., & Pohmann, R. (2023). Concurrent intrinsic optical imaging and fMRI at ultra-high field using magnetic field proof optical components. *NMR in Biomedicine*, *36*(7), e4909. https://doi.org/10.1002/nbm.4909

Blinder, P., Tsai, P. S., Kaufhold, J. P., Knutsen, P. M., Suhl, H., & Kleinfeld, D. (2013). The cortical angiome: An interconnected vascular network with noncolumnar patterns of blood flow. *Nature Neuroscience*, *16*(7), Article 7. https://doi.org/10.1038/nn.3426

Boas, D. A., Jones, S. R., Devor, A., Huppert, T. J., & Dale, A. M. (2008). A vascular anatomical network model of the spatio-temporal response to brain activation. *NeuroImage*, *40*(3), Article 3. https://doi.org/10.1016/j.neuroimage.2007.12.061

Buck, J., Larkin, J. R., Simard, M. A., Khrapitchev, A. A., Chappell, M. A., & Sibson, N. R. (2018). Sensitivity of Multiphase Pseudocontinuous Arterial Spin Labelling (MP pCASL) Magnetic Resonance Imaging for Measuring Brain and Tumour Blood Flow in Mice. *Contrast Media & Molecular Imaging*, *2018*, 1–11. https://doi.org/10.1155/2018/4580919

Buxton, R. B. (2001). The elusive initial dip. *Neuroimage*, *13*(6 Pt 1), 953–958. https://doi.org/10.1006/nimg.2001.0814

Buxton, R. B., Uludağ, K., Dubowitz, D. J., & Liu, T. T. (2004). Modeling the hemodynamic response to brain activation. *NeuroImage*, *23*, S220–S233. https://doi.org/10.1016/j.neuroimage.2004.07.013

Christen, T., Zaharchuk, G., Pannetier, N., Serduc, R., Joudiou, N., Vial, J.-C., Rémy, C., & Barbier, E. L. (2012). Quantitative MR estimates of blood oxygenation based on T2*: A numerical study of the impact of model assumptions. *Magnetic Resonance in Medicine*, *67*(5), 1458–1468.

Desjardins, M., Kılıç, K., Thunemann, M., Mateo, C., Holland, D., Ferri, C. G., Cremonesi, J. A., Li, B., Cheng, Q., & Weldy, K. L. (2019). Awake mouse imaging: From two-photon microscopy to blood oxygen level–dependent functional magnetic resonance imaging. *Biological Psychiatry: Cognitive Neuroscience and Neuroimaging*, *4*(6), 533–542. https://doi.org/10.1016/j.bpsc.2018.12.002

Fang, Q., Sakadžić, S., Ruvinskaya, L., Devor, A., Dale, A. M., & Boas, D. A. (2008). Oxygen Advection and Diffusion in a Three Dimensional Vascular Anatomical Network. *Optics Express*, *16*(22), Article 22. https://doi.org/10.1364/oe.16.017530

Gagnon, L., Sakadžić, S., Lesage, F., Musacchia, J. J., Lefebvre, J., Fang, Q., Yücel, M. A., Evans, K. C., Mandeville, E. T., Cohen-Adad, J., Polimeni, J. R., Yaseen, M. A., Lo, E. H., Greve, D. N., Buxton, R. B., Dale, A. M., Devor, A., & Boas, D. A. (2015). Quantifying the microvascular origin of BOLD-fMRI from first principles with two-photon microscopy and an oxygen-sensitive nanoprobe. *Journal of Neuroscience*, *35*(8), Article 8. https://doi.org/10.1523/JNEUROSCI.3555-14.2015

Gertz, K., Priller, J., Kronenberg, G., Fink, K. B., Winter, B., Schröck, H., Ji, S., Milosevic, M., Harms, C., Böhm, M., Dirnagl, U., Laufs, U., & Endres, M. (2006). Physical Activity Improves Long-Term Stroke Outcome via Endothelial Nitric Oxide Synthase–Dependent Augmentation of Neovascularization and Cerebral Blood Flow. *Circulation Research*, *99*(10), Article 10. https://doi.org/10.1161/01.RES.0000250175.14861.77

Gould, I. G., Tsai, P., Kleinfeld, D., & Linninger, A. (2017). The capillary bed offers the largest hemodynamic resistance to the cortical blood supply. *Journal of Cerebral Blood Flow & Metabolism*, *37*(1), Article 1. https://doi.org/10.1177/0271678X16671146

Griffeth, V. E. M., & Buxton, R. B. (2011). A theoretical framework for estimating cerebral oxygen metabolism changes using the calibrated-BOLD method: Modeling the effects of blood volume distribution, hematocrit, oxygen extraction fraction, and tissue signal properties on the BOLD signal. *NeuroImage*, *58*(1), Article 1. https://doi.org/10.1016/j.neuroimage.2011.05.077

Hartung, G., Badr, S., Mihelic, S., Dunn, A., Cheng, X., Kura, S., Boas, D., Kleinfeld, D., Alaraj, A., & Linninger, A. (2021). Mathematical synthesis of the cortical circulation for the whole mouse brain-Part II: microcirculatory closure. *Computers in Biology and Medicine*, e12687–e12687. https://doi.org/10.1111/micc.12687

Hartung, G., Badr, S., Moeini, M., Lesage, F., Kleinfeld, D., Alaraj, A., & Linninger, A. (2021). Voxelized simulation of cerebral oxygen perfusion elucidates hypoxia in aged mouse cortex. *PLoS Computational Biology*, *17*(1), e1008584. https://doi.org/10.1371/journal.pcbi.1008584

Hill, A. V. (1910). The possible effects of the aggregation of the molecules of haemoglobin on its dissociation curves. *J. Physiol.*, *40*, 4–7.

Le, T. T., Im, G. H., Lee, C. H., Choi, S. H., & Kim, S.-G. (2024). Mapping cerebral perfusion in mice under various anesthesia levels using highly sensitive BOLD MRI with transient hypoxia. *Science Advances*, *10*(9), eadm7605. https://doi.org/10.1126/sciadv.adm7605

Lee, D., Le, T. T., Im, G. H., & Kim, S.-G. (2022). Whole-brain perfusion mapping in mice by dynamic BOLD MRI with transient hypoxia. *Journal of Cerebral Blood Flow & Metabolism*, *42*(12), 2270–2286. https://doi.org/10.1177/0271678X221117008

Linninger, A. A., Gould, I. G., Marinnan, T., Hsu, C.-Y., Chojecki, M., & Alaraj, A. (2013). Cerebral Microcirculation and Oxygen Tension in the Human Secondary Cortex. *Annals of Biomedical Engineering*, *41*(11), Article 11. https://doi.org/10.1007/s10439-013-0828-0

Linninger, A., Hartung, G., Badr, S., & Morley, R. (2019). Mathematical synthesis of the cortical circulation for the whole mouse brain-part I: theory and image integration. *Computers in Biology and Medicine*, *110*, 265–275. https://doi.org/10.1016/j.compbiomed.2019.05.004

Maeda, K., Mies, G., Oláh, L., & Hossmann, K.-A. (2000). Quantitative Measurement of Local Cerebral Blood Flow in the Anesthetized Mouse Using Intraperitoneal [14C]Iodoantipyrine Injection and Final Arterial Heart Blood Sampling. *Journal of Cerebral Blood Flow & Metabolism*, *20*(1), Article 1. https://doi.org/10.1097/00004647-200001000-00003

Mandeville, J. B., Marota, J. J., Ayata, C., Zaharchuk, G., Moskowitz, M. A., Rosen, B. R., & Weisskoff, R. M. (1999). Evidence of a cerebrovascular postarteriole windkessel with delayed compliance. *Journal of Cerebral Blood Flow & Metabolism*, *19*(6), Article 6. https://doi.org/10.1097/00004647-199906000-00012

Mintun, M. A., Lundstrom, B. N., Snyder, A. Z., Vlassenko, A. G., Shulman, G. L., & Raichle, M. E. (2001). Blood flow and oxygen delivery to human brain during functional activity: Theoretical modeling and experimental data. *PNAS*, *98*(12), Article 12. https://doi.org/10.1073/pnas.111164398

Moeini, M., Lu, X., Avti, P. K., Damseh, R., Bélanger, S., Picard, F., Boas, D., Kakkar, A., & Lesage, F. (2018). Compromised microvascular oxygen delivery increases brain tissue vulnerability with age. *Scientific Reports*, *8*(1), Article 1. https://doi.org/10.1038/s41598-018-26543-w

Munting, L. P., Derieppe, M., Suidgeest, E., Hirschler, L., Van Osch, M. J., Denis de Senneville, B., & van Der Weerd, L. (2021). Cerebral blood flow and cerebrovascular reactivity are preserved in a mouse model of cerebral microvascular amyloidosis. *Elife*, *10*, e61279. https://doi.org/10.7554/eLife.61279

Pathak, A. P., Ward, B. D., & Schmainda, K. M. (2008). A novel technique for modeling susceptibility-based contrast mechanisms for arbitrary microvascular geometries: The finite perturber method. *NeuroImage*, *40*(3), 1130–1143. https://doi.org/10.1016/j.neuroimage.2008.01.022

Sakadžić, S., Mandeville, E. T., Gagnon, L., Musacchia, J. J., Yaseen, M. A., Yucel, M. A., Lefebvre, J., Lesage, F., Dale, A. M., Eikermann-Haerter, K., Ayata, C., Srinivasan, V. J., Lo, E. H., Devor, A., & Boas, D. A. (2014). Large arteriolar component of oxygen delivery implies a safe margin of oxygen supply to cerebral tissue. *Nature Communications*, *5*, 5734. https://doi.org/10.1038/ncomms6734

Sakadžić, S., Yaseen, M. A., Jaswal, R., Roussakis, E., Dale, A. M., Buxton, R. B., Vinogradov, S. A., Boas, D. A., & Devor, A. (2016). Two-photon microscopy measurement of cerebral metabolic rate of oxygen using periarteriolar oxygen concentration gradients. *Neurophotonics*, *3*(4), 045005. https://doi.org/10.1117/1.NPh.3.4.045005

Şencan, İ., Esipova, T., Kılıç, K., Li, B., Desjardins, M., Yaseen, M. A., Wang, H., Porter, J. E., Kura, S., Fu, B., & others. (2022). Optical measurement of microvascular oxygenation and blood flow responses in awake mouse cortex during functional activation. *Journal of Cerebral Blood Flow & Metabolism*, *42*(3), 510–525. https://doi.org/10.1177/0271678X20928011

Tian, P., Teng, I. C., May, L. D., Kurz, R., Lu, K., Scadeng, M., Hillman, E. M. C., Crespigny, A. J. D., D'Arceuil, H. E., Mandeville, J. B., Marota, J. J. A., Rosen, B. R., Liu, T. T., Boas, D. A., Buxton, R. B., Dale, A. M., & Devor, A. (2010). Cortical depth-specific microvascular dilation underlies laminar differences in blood oxygenation level-dependent functional MRI signal. *Proceedings of the National Academy of Sciences*, *107*(34), Article 34. https://doi.org/10.1073/pnas.1006735107

Truskey, G., Yuan, F., & Katz, D. (2004). *Transport Phenomena in Biological Systems*. Pearson Prentice Hall.

Uchida, K., Reilly, M. P., & Asakura, T. (1998). Molecular stability and function of mouse hemoglobins. *Zoological Science*, *15*(5), 703–706. https://doi.org/10.2108/zsj.15.703

Uludağ, K., Müller-Bierl, B., & Uğurbil, K. (2009). An integrative model for neuronal activity-induced signal changes for gradient and spin echo functional imaging. *NeuroImage*, *48*(1), Article 1. https://doi.org/10.1016/j.neuroimage.2009.05.051

Vazquez, A. L., Fukuda, M., Tasker, M. L., Masamoto, K., & Kim, S.-G. (2010). Changes in cerebral arterial, tissue and venous oxygenation with evoked neural stimulation: Implications for hemoglobin-based

functional neuroimaging. *Journal of Cerebral Blood Flow & Metabolism*, *30*(2), 428–439. https://doi.org/10.1038/jcbfm.2009.213

Xu, K., Radhakrishnan, K., Serhal, A., Allen, F., LaManna, J. C., & Puchowicz, M. A. (2011). Regional Brain Blood Flow in Mouse: Quantitative Measurement Using a Single-Pass Radio-Tracer Method and a Mathematical Algorithm. In J. C. LaManna, M. A. Puchowicz, K. Xu, D. K. Harrison, & D. F. Bruley (Eds.), *Oxygen Transport to Tissue XXXII* (pp. 255–260). Springer US.

Zhu, X.-H., Zhang, Y., Tian, R.-X., Lei, H., Zhang, N., Zhang, X., Merkle, H., Ugurbil, K., & Chen, W. (2002). Development of ^{17}O NMR approach for fast imaging of cerebral metabolic rate of oxygen in rat brain at high field. *Proceedings of the National Academy of Sciences*, *99*(20), 13194–13199. https://doi.org/10.1073/pnas.202471399
